# The impact of cellular senescence in human adipose tissue

**DOI:** 10.1007/s12079-023-00769-4

**Published:** 2023-05-17

**Authors:** Annika Nerstedt, Ulf Smith

**Affiliations:** Lundberg Laboratory for Diabetes Research, Department of Molecular and Clinical Medicine, Sahlgrenska University Hospital, Sahlgrenska Academy, University of Gothenburg, Blå Stråket 5, SE-413 45 Gothenburg, Sweden

**Keywords:** Cellular senescence, Adipose tissue, Obesity, Insulin resistance, Type 2 diabetes, Inflammation

## Abstract

In the last decades the prevalence of obesity has increased dramatically, and the worldwide epidemic of obesity and related metabolic diseases has contributed to an increased interest for the adipose tissue (AT), the primary site for storage of lipids, as a metabolically dynamic and endocrine organ. Subcutaneous AT is the depot with the largest capacity to store excess energy and when its limit for storage is reached hypertrophic obesity, local inflammation, insulin resistance and ultimately type 2 diabetes (T2D) will develop. Hypertrophic AT is also associated with a dysfunctional adipogenesis, depending on the inability to recruit and differentiate new mature adipose cells. Lately, cellular senescence (CS), an aging mechanism defined as an irreversible growth arrest that occurs in response to various cellular stressors, such as telomere shortening, DNA damage and oxidative stress, has gained a lot of attention as a regulator of metabolic tissues and aging-associated conditions. The abundance of senescent cells increases not only with aging but also in hypertrophic obesity independent of age. Senescent AT is characterized by dysfunctional cells, increased inflammation, decreased insulin sensitivity and lipid storage. AT resident cells, such as progenitor cells (APC), non-proliferating mature cells and microvascular endothelial cells are affected with an increased senescence burden. Dysfunctional APC have both an impaired adipogenic and proliferative capacity. Interestingly, human mature adipose cells from obese hyperinsulinemic individuals have been shown to re-enter the cell cycle and senesce, which indicates an increased endoreplication. CS was also found to be more pronounced in mature cells from T2D individuals, compared to matched non-diabetic individuals, with decreased insulin sensitivity and adipogenic capacity.

**Graphical abstract:**

Factors associated with cellular senescence in human adipose tissue
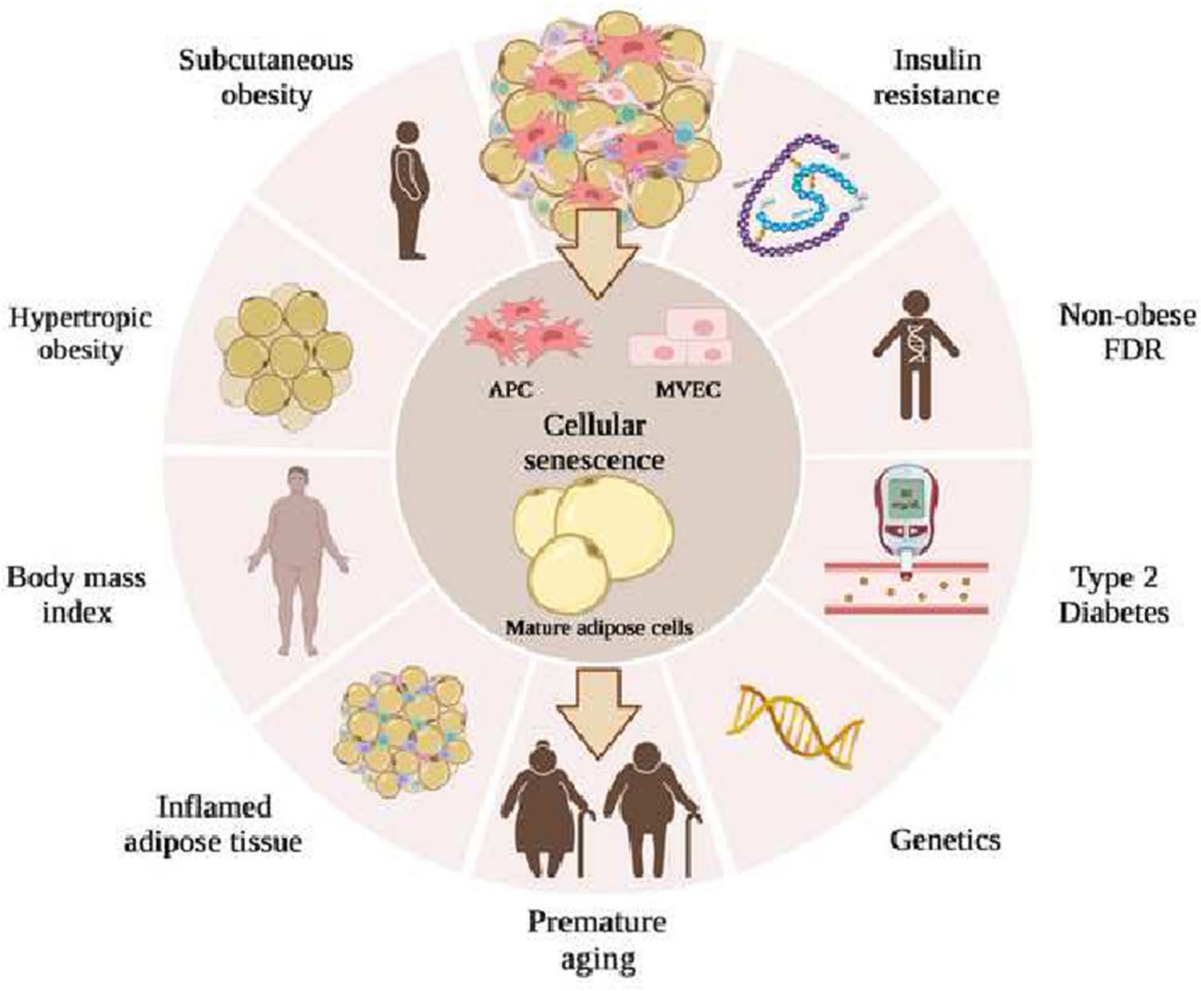

## Introduction

Due to the worldwide epidemic of obesity and type 2 diabetes (T2D), the adipose tissue (AT) biology and physiology has received increased attention. Nowadays, AT has gone from being only considered as a fat storing tissue to a highly active metabolic and endocrine organ with impact on the whole-body (Ottaviani et al. 2011). However, AT is still the primary site for accumulation of excess energy to be used whenever the demands arise. Not only lipogenesis is of importance also lipolysis has to be tightly regulated and both obesity and T2D could have detrimental effects on adipose tissue metabolism (Singla et al. 2010).

Cellular senescence (CS) is induced as a stress response and defined as an irreversible cell cycle arrest with antitumorigenic effects. Senescent cells cease to proliferate but are still metabolic active and they adopt specific characteristics, with increased cellular growth and secretion of various factor called the senescence-associated secretory phenotype (SASP). Senescent cells have also impact on several other processes, such as wound healing, fibrosis and aging. Lately, CS has also gained a lot of attention due to its involvement in the development and progression of metabolic disorders, such as obesity and T2D (Burton et al.).

## The importance of a healthy adipose tissue

### Adipose tissue depots and resident cells

AT is distributed throughout the whole body and divided into the main adipose depots, subcutaneous AT (SAT), located under the skin, and visceral AT (VAT), within the abdominal wall and surrounding the internal organs (Shen et al. 2003). SAT is the largest adipose depot and stores about 80% of total body fat and VAT constitutes around 10–20% (Janochova et al. 2019) and for both SAT and VAT the depot of interest is found in the abdomen. Increased VAT is well known to associate with metabolic complication, such as insulin resistance (IR), increased risk for T2D and cardiovascular disease (CVD).

AT is composed of a wide variety of cell types, mature adipose cells of various size, adipose tissue derived progenitor cells (APC), microvascular endothelia cells (MVEC), and different immune cells. Even though mature adipose cells only account for 20–40% of AT resident cells they occupy more or less the whole tissue (Eto et al. 2009). The size of mature adipose cells has been widely used as an important factor for relating cellular functions and pathophysiological conditions.

### The size of mature adipose cells as a predictor of metabolic disease

**Fig. 1:**
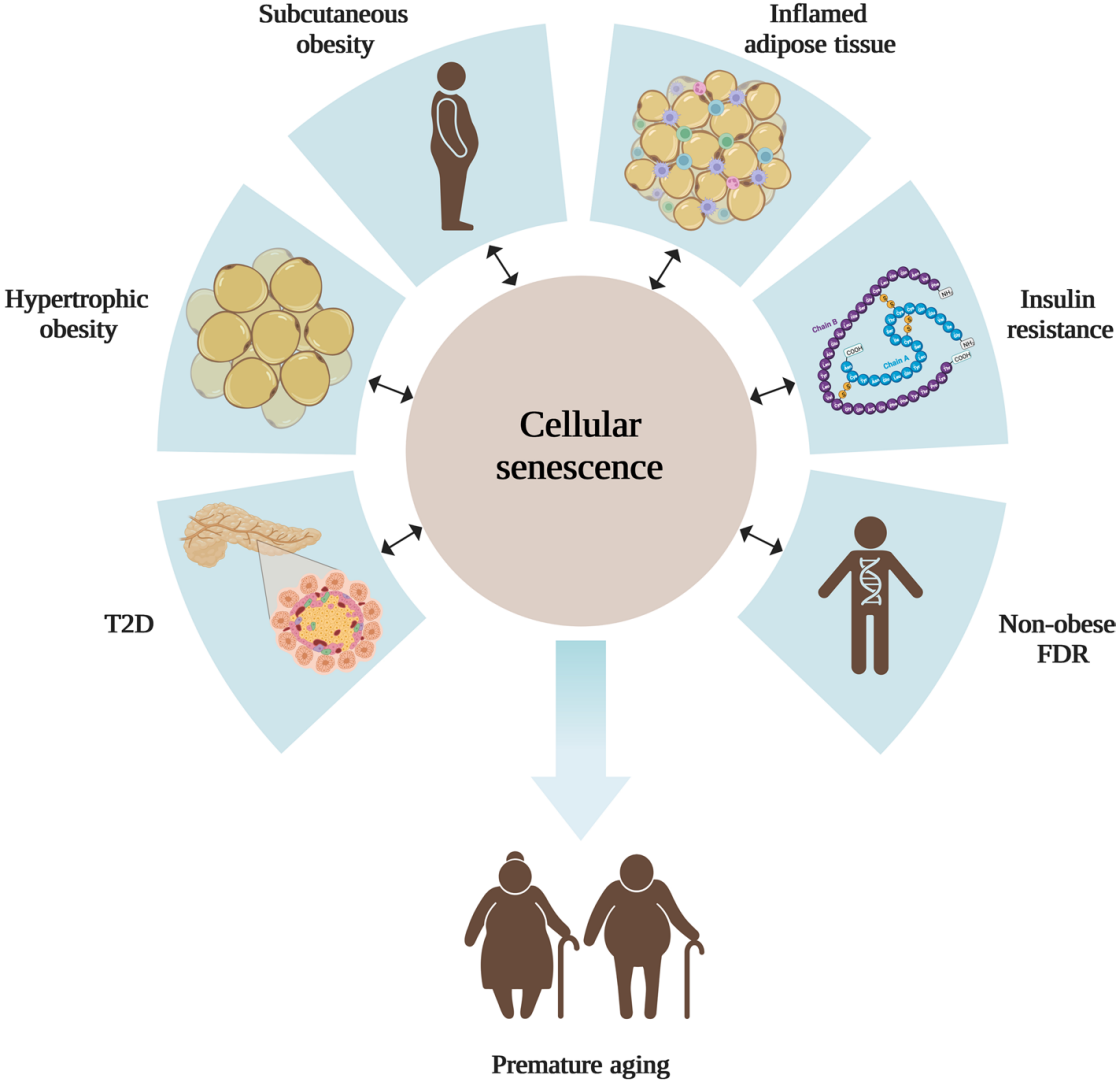
Cellular senescence and its association with metabolic complications in obesity. Cellular senescence is an irreversible state of cell cycle arrest and has been implicated as a regulator of metabolic tissues, such as subcutaneous adipose tissue (SAT), and as a contributor to aging-associated conditions. SAT is the preferred adipose depot for fat accumulation, but when it reaches its limitation for storing lipids hypertrophic obesity, insulin resistance and ultimately T2D will occur. Chronic low-grade inflammation, due to a dysfunctional SAT, with increased infiltration of inflammatory macrophages will contribute and exacerbate these age-related conditions. Also, non-obese FDR, individuals with genetic predisposition for T2D, has a phenotype resembling premature aging. Abbreviations: FDR, first-degree relatives of type 2 diabetics; T2D, Type 2 Diabetes. Figure created with Biorender.com

Healthy AT expands through a combination of hypertrophy (enlargement of mature adipose cells) and hyperplasia (recruitment and differentiation of APC). Mature adipose cell growth per se is a necessary mechanism to be able to accommodate excess nutrients and store as triglycerides. SAT is the depot that has the largest capacity to store lipids and, if needed, recruiting new APC will protect against metabolic disease by preventing hypertrophic expansion (Gustafson et al. 2015b; Laforest et al. 2015). However, when SAT has reached its limitation for expansion hypertrophic obesity, local inflammation and IR will emerge (Neeland et al. 2012; Weisberg et al 2003) (Fig. 1).The size of mature adipose cells is influenced by obesity, gender and anatomical localization, and varies considerably in cell diameter, from 20 μm to several hundred μm in humans (Fang et al. 2015; Kloting et al. 2010). Based on a recent publication it was shown that the mean diameter of mature adipose cells in SAT from lean individuals was 75–80 μm and that it increased with body mass index (BMI) and reached a plateau at 120 μm in extremely obese subjects (Ye et al. 2022).

Obesity is known to be a major risk factor for T2D (Kahn and Flier 2000) and visceral obesity has been associated with a higher risk of metabolic disease compared to subcutaneous obesity with little or no risk or even being protective (Tchkonia et al. 2010). Adipose cell hypertrophy has been shown to be more highly associated with IR, lipid metabolism, T2D diabetes and cardiovascular disorders than the effect of obesity alone (Ye et al. 2022) (Fig. 1).

A positive correlation between SAT adipose cell size and IR, independent of BMI in non-diabetic individuals has been found. However, this was not seen in T2D individuals, which suggests that other factors, such as free fatty acids and glucose, adds to the effect of adipose cell size (Lundgren et al. 2007). Furthermore, in a cohort of individual, highly variable in BMI, adipose cell size was positively correlated with IR, measured as HOMA index, and insulin levels (Arner et al. 2010). It has also been shown that IR individuals, compared to BMI-matched insulin-sensitive individuals, are characterized by enlargement of large mature adipose cells, a larger proportion of small adipose cells together with decreased expression of the differentiation markers, *peroxisome proliferator-activated receptor g 2* (*PPARG2*), *solute carrier family 2 member 4* (also known as *GLUT4*), and *adiponectin*, which suggests an impaired adipogenesis (McLaughlin et al. 2007, 2014). This was also shown in T2D individuals compared to obese individuals with the same BMI (Pasarica et al. 2009). Further, in young and non-obese first-degree relatives of type 2 diabetics (FDR), increased IR correlated with inappropriately expanded adipose cells for their BMI, which was not seen in matched individuals with family predisposition for obesity (Yang et al. 2012). This was subsequently documented in a large group of FDR independent of obesity (Arner et al. 2011). It has also been demonstrated in populations with different ethnicity that enlarged SAT mature adipose cells are associated with IR and could predict the incidence of T2D independent of body fat and BMI (Weyer et al. 2000; Lonn et al. 2010). In a recent study, adipose cell hypertrophy was found to be associated with telomere shortening, as a marker of biological or premature aging, low levels of adiponectin and increased oxidative stress in obese and T2D individuals (Monickaraj et al. 2012). Taken together, adipose cell hypertrophy, independent of obesity and BMI, is associated with IR and predicts T2D, not only in non-obese individuals of different ethnicity but also in non-obese individuals with a genetic predisposition for T2D (Fig. 1). SAT adipose cell hypertrophy also suggests an impaired adipogenesis due to an inability to recruit and differentiate new mature adipose cells (Gustafson et al. 2013). However, hypertrophic obesity is also associated with an increased lipolysis, which is further aggravated by an impaired anti-lipolytic effect of insulin (Smith and Kahn 2016).

### Obesity, insulin resistance and chronic low-grade inflammation

Numerous studies have identified a close association between obesity, IR and chronic low-grade inflammation (Hammarstedt et al. 2018) (Fig. 1) and both human and animal studies have shown that AT is the primary site for the initiation and aggravation of obesity-related chronic low-grade inflammation (Weisberg et al. 2003; Xu et al. 2003). However, it is not an increased fat mass per se but rather a dysfunctional SAT with hypertrophic mature adipose cells that drives the induction of chronic low-grade inflammation and IR in metabolically unhealthy subjects. This remodeling of AT triggers the activation of the mitogen-activated protein kinase 8 (also known as JNK1) and nuclear factor kappa B (NF-*k*B) signaling pathways, which have negative impacts on insulin signaling as well as increased expression of several proinflammatory genes. This also increases the infiltration and activation of inflammatory macrophages, known to produce and secrete chemokines and proinflammatory cytokines, such as interleukin 6 (IL6) and tumor necrosis factor-α (TNFα), where both have been shown to directly inhibit insulin signaling (Zatterale et al. 2019).

A dysfunctional AT not only adopts a proinflammatory phenotype that is harmful for microenvironment, the increased storage of free fatty acids leads also to increased lipolysis, enhanced endoplasmic reticulum and oxidative stress, and mitochondrial and lysosomal dysfunction (Ahmed et al. 2021). Lately, these complications due to a hypertrophic AT has also been suggested to be associated with CS (Narasimhan et al. 2021), which will be discussed later in this review (Fig. 1).

### Hypertrophic obesity and adipogenesis

For a more detailed review of adipogenesis, which is out of scope in this article, we refer to our recent publication by Hammarstedt et al. (2018). Adipogenesis is a highly complex process, which starts with the recruitment and commitment of APC into preadipocytes followed by terminal differentiation into mature adipose cells. Before entering the final differentiation step the recruited APC have to undergo mitotic clonal expansion, which is regulated by the bone morphogenetic protein 4 (BMP4) (Bowers et al. 2006). BMP4 dissociates a differentiation inhibitory complex, including the transcriptional coactivator zinc finger protein 423 (ZNF423). ZNF423 will enter the nucleus and activate the transcription of *PPARG* (Hammarstedt et al. 2013), the master regulator of adipogenic differentiation together with the transcription factor *CCAAT enhancer binding protein alpha* (Wu et al. 1999). This allows the cells to mature with the ability to process and store lipids, secrete adipokines, such as adiponectin (known to enhance insulin sensitivity in peripheral tissues (Turer and Scherer 2012)) and BMP4, respond to insulin and to maintain the phenotype of mature adipose cells. However, the effect of BMP4 is strongly regulated by BMP inhibitors, such as Gremlin 1 (Topol et al. 2000).

As mentioned above a decreased adipogenic potential has been seen in hypertrophic obesity. However, it is not the number of human subcutaneous APC per se that is reduced, instead it is the number or cells that has the capacity to undergo differentiation that is decreased (Gustafson et al. 2019). In hypertrophic obesity the phenotype is changed, and the adipose cells become IR, which is associated with a proinflammatory profile with increased levels of cytokines and chemokines as well as increased infiltration of macrophages. These macrophages further promote the inflammatory phenotype including secretion of TNFα (Gustafson et al. 2009). Furthermore, TNFα has been shown to completely inhibit the adipogenic potential of APC and to induce a macrophage-like phenotype in these cells (Isakson et al. 2009).

## Cellular senescence in adipose tissue

### Overview

Replicative senescence was first described by Hayflick and Moorhead in cultured human fibroblasts. Upon repeated serial passing these cells showed a limited replicative capacity and become irreversibly arrested, which was considered as a hallmark of aging (Hayflick and Moorhead 1961). Nowadays, senescence is defined as a process where cells age and become permanently cell cycle arrested but still are alive, metabolically active and resistant to apoptosis (Kumari and Jat 2021). CS is a highly dynamic process driven by both tissue- and context-specific effects as well as epigenetic and genetic changes (van Deursen 2014). There are two main types of senescence, replicative senescence, as previously mentioned, and stress-induced premature senescence (SIPS). SIPS can be induced by variety of stressors, such as oxidative stress, metabolic dysfunction, epigenetic alteration, radiation, endoplasmic reticulum (ER) stress, genotoxic drugs as well as mechanical and shear stress (Gorgoulis et al. 2019). However, depending on the inducer of CS, different pathways and factors will be affected leading to DNA damage, telomere shortening, inflammation, mitochondrial dysfunction, hypoxia, oncogene activation and increased secretion of cytokines, chemokines, proteases and growth factors, known as the senescence-associated secretory phenotype (SASP) and decreased cell differentiation (Stout et al. 2017; Gustafson et al. 2019; Gorgoulis et al. 2019). Specific SASP factors vary depending on cell and tissue origin but also on the inducers of senescence. However, IL6 and IL8 are mostly found to be present (Coppe et al. 2010) and TNFα is also secreted by a large variety of cells (Kandhaya-Pillai et al. 2017). SASPs act through both autocrine, by re-enforcing CS, and paracrine factors, by inducing senescence in neighboring non-senescent cells which further contributes to the age-related disorders (Acosta et al. 2013). Taken together, due to secreted SASP factors senescent cell can be a source for the low-grade chronic inflammation seen in the microenvironment of a dysfunctional AT. However, CS has not only detrimental effects it has also certain beneficial effects on embryogenesis, wound healing and tumor suppression (Gorgoulis et al. 2019).

Senescent cells undergo changes in morphology and senescence-associated proteins. In culture, these cells appear to be larger, flattened, have more granules in the cytoplasm, multinucleated, more vacuoles and the nucleus shows signs of DNA damage with increased phosphorylation of the H2A histone family X (γH2AX) (Beck et al. 2020). Furthermore, senescent cells have disrupted nuclear membrane due to decreased expression of *lamin B1* (*LMNB1*), a key trigger of global chromatin reconfiguration, as well as chromatin reorganization (Liu et al. 2020). Also, the lysosomal compartment is largely increased in senescent cells, which is reflected by an increased activity of the characteristic senescence-associated β-galactosidase (SA-β-Gal) (Rovira et al. 2022).

To establish a senescent cell-cycle arrest, due to an unresolved DNA damage, the activation of p53 (encoded by *tumor protein p53* (*TP53*)) and/or retinoblastoma (RB) tumor suppressor pathways play a central role, which leads to the accumulation of the cyclin dependent kinase (CDK) 2 inhibitor p21 (encoded by cyclin dependent kinase inhibitor (*CDKN*) *1A*) and CDK4/6 inhibitor p16 (encoded by *CDKN2A*). As a consequence of this, RB will be activated and result in a persistent cell-cycle arrest (Gorgoulis et al. 2019). One important feature of senescent cells is to avoid apoptosis and resist self-clearance, which they do by activating a network of antiapoptotic senescent cell pathways (SCAPs), such as B-cell lymphoma (BCL) 2/BCL-extra large (XL), phosphatidylinositol 4,5-bisphosphate 3-kinase (PI3K)/ serine/threonine kinase 1 (AKT), or p53/p21/serpine (Kirkland and Tchkonia 2017). Since both senescence and apoptosis trigger the p53 pathway it has been suggested that the level and activity of p53, are of most importance for senescent cells to resist apoptosis and to enter a permanent cell cycle arrest (Kirschner et al. 2015). Even though senescent cells share common characteristics, these do not always occur at the same time and intensity, making the senescence phenotype extremely dynamic and complex. Therefore, it is of major importance to use multiple markers to determine if the cells have adopted a senescent phenotype (Liu et al. 2020).

Both CS and SASP are potential therapeutic targets and senotherapeutics, a new class of drugs and natural products targeting CS, is under development. Senotherapeutics consist of two members, senomorphics and senolytics. Senolytics act directly on the senescent cells by selectively killing them through apoptosis. On the contrary, senomorphic compounds act, without killing senescent cells, on pathologic SASP signaling pathways by preventing the production of, neutralizing or antagonizing SASP factors (Lagoumtzi and Chondrogianni 2021).

### Cellular senescence and adipose tissue

AT is an endocrine organ known to be important for maintenance of lipid and energy homeostasis as well as systemic glucose levels. However, these functions decrease with aging and obesity. It is well known that aging is a major risk factor for many chronic states/diseases, including IR, T2D, nonalcoholic fatty liver disease (NAFLD), and CVD (Tchkonia and Kirkland 2018). Many of these chronic diseases show a state of premature or accelerated aging. Biological aging could be defined as a state of replicative senescence and, lately, much attention has been paid to CS as a regulator of metabolic tissues and as a contributor to aging-associated conditions. Recently, the Tabula Muris study showed an age-associated increase in tissue/CS in mice contributing to dysfunctional cells, increased inflammation and accumulation of markers of genetic instability (Tabula Muris 2020). Furthermore, AT is one of the most vulnerable tissues when it comes to aging (Ou et al. 2022) and also a tissue where CS according to an animal study is initiated most early (Tabula Muris 2020). The two main adipose depots*,* SAT and VAT, are differentially associated with the clinical outcomes of obesity and aging as discussed above. It has also been shown that the telomere length, as a marker of risk of CS, is shorter in SAT than in VAT suggesting that SAT is more prone to age-related injuries (Lakowa et al. 2015).

### Cellular senescence and its consequences for adipogenesis

There is a need for an annual renewal of 10% of mature adipose cells in adulthood (Spalding et al. 2008) and to maintain the functionality of the AT, new cells has to be recruited into the adipogenic program. The first step is to recruit APC, which are committed into preadipocytes followed by the second step of differentiation to become mature adipose cells. CS has a high impact on AT with a dysfunctional adipogenesis as result.

**Fig. 2:**
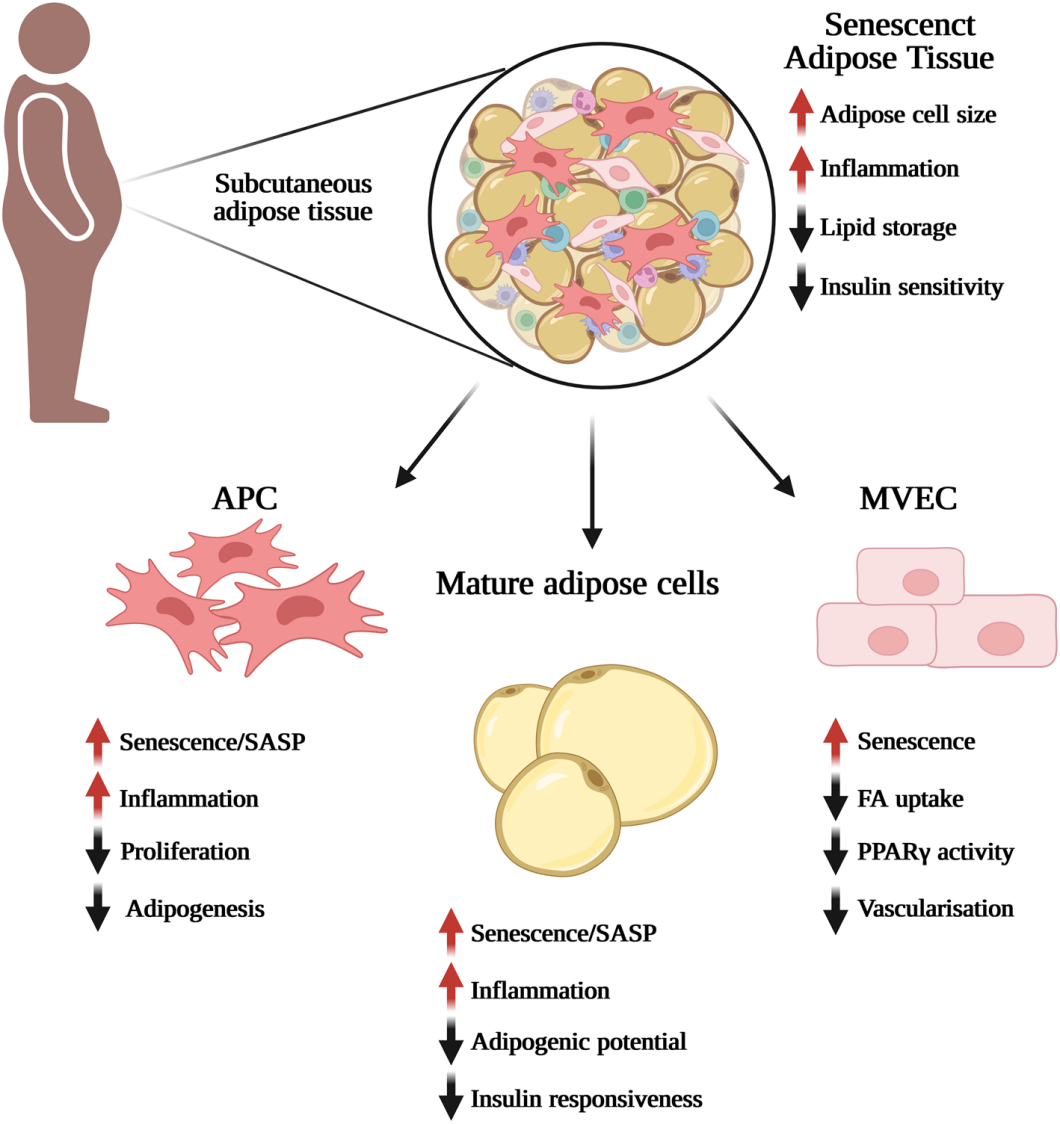
Cellular senescence and its effect on adipose tissue resident cells. Adipose tissue and its resident cells are highly susceptible to age-related complications and cellular senescence has various harmful effects on their functions. The morphology of the cells changes, the expression of senescence markers increases together with SASP acquisition. Adipose tissue-derived progenitor cells show decreased proliferation and a dysfunctional adipogenesis. The phenotype for mature adipose cells displays a decreased adipogenic potential and insulin sensitivity together with increased endoreplication. Decreased fatty acid uptake and PPARg activity as well as dysfunctional vascularization is seen in microvascular endothelial cells. Abbreviations: APC, adipose tissue derived progenitor cells; FA, fatty acid; MVEC, microvascular endothelial cells; PPARγ, peroxisome proliferator-activated receptor γ; SASP, senescence-associated secretory phenotype. Figure created with Biorender.com

In earlier studies we have shown that the adipogenic potential of APC from human SAT has a high inter-individual variability and that markers for reduced adipogenesis, such as PPARγ and reduced lipid accumulation, reduced insulin sensitivity and increased inflammation correlate with hypertrophic obesity and genetic predisposition for T2D in FDR (Gustafson et al. 2013, 2015a, 2015b; Arner et al. 2011). However, both the proliferative as well as the differentiation capacity of senescent APC are reduced (Xu et al. 2015b; Mitterberger et al. 2014; Gustafson et al. 2019) (Fig. 2). More recently, we have shown that the number of human subcutaneous APC in hypertrophic obesity and T2D are not reduced, instead these cells have a dysfunctional adipogenesis due to increased CS with high levels of p53 and p16 (Gustafson et al. 2019). Since p53 has a central role in the induction of CS and has to be downregulated to allow the adipogenic program to progress, this could be one of the reasons for the impaired adipogenesis. It was also shown that human senescent APC cells secrete activin A, a SASP factor known to affect adipogenesis negatively, which rendered non-senescent APC senescent and suppressed their adipogenic capacity (Xu et al. 2015b). In the same study senescent cells, in 18-month-old mice, were targeted by janus kinase (JAK) inhibitors, which blunted activin A and increased adipogenesis (Xu et al. 2015b). Not only senescent cells per se but also the paracrine effect of secreted SASP factors have a massive impact on the adipogenic potential of neighboring non-senescent APC (Fig. 2).

### Cellular senescence in adipose tissue derived progenitor cells (APC)

Not only aging, also the onset of obesity is linked to an increased burden of senescent cells within the adipose tissue, and it has been reported that APC can senesce due to replicative senescence in aging or by increased oxidative stress in obesity (Xu et al. 2015a; Mitterberger et al. 2014).

In an early study, a link between increased p53 expression in AT and obesity, senescence, and age-related metabolic disorders was found (Minamino et al. 2009). Consistent with this, APC from obese individuals exhibited a limited replicative potential as well as increased expression of senescence markers, *TP53, CDKN1A*, *CDKN2A*, *IL6* and *C–C motif chemokine ligand 2* (*CCL2*) compared to lean individuals (Conley et al. 2020). Also, in subcutaneous APC from young obese mice increased levels of p21 and p16 were detected (Alessio et al. 2020). Furthermore, it was shown in human SAT that the senescence markers, *TP53,* *galactosidase beta 1 *(*GLB1*) and *serpin family E member 1* (*SERPINE1*)*,* were increased in hypertrophic obesity and further increased in similarly obese T2D individuals. For APC to enter the adipogenic program p53 must be downregulated, which turned out to be impaired in human senescent APC, as discussed above (Gustafson et al. 2019). This was also valid for APC from FDR, suggesting that increased APC senescence is associated with reduced adipogenesis before the onset of T2D (Gustafson et al. 2019).

Comparing a panel of senescence markers in APC from FDR and control individuals, increased number of SA-β-gal and p21 positive cells together with increased number of cells arrested in the G_1_ phase of the cell cycle was found as well as increased levels of secreted SASPs (Spinelli et al. 2022). It is well known that senescent APC affect neighboring non-senescent APC via secreted SASP factors, such as activin A, IL6 and TNFα contributing to impaired adipogenesis as well as reduced insulin sensitivity (Zaragosi et al. 2010; Xu et al. 2015a, 2015b) and also likely involved in the development of T2D. Furthermore, altered DNA methylation is a powerful epigenetic modification that has been implicated in the development of CS (Crouch et al. 2022). In a study characterizing the methylome in APC from FDR and matched control individuals it was found that APC from FDR had several hypomethylated genes related to senescence with *zinc finger matrin-type 3* (*ZMAT3*) as one of the top-ranked genes (Parrillo et al. 2020). *ZMAT3* hypomethylation in APC from FDR leads to increased levels of ZMAT3 and p53 as well as induced expression of *CDKN1A* and consequently premature senescence (Spinelli et al. 2022). It was also shown that ZMAT3 interacts with the p53/p21 pathway in APC to induce senescence. Furthermore, in vitro overexpression of ZMAT3 in human APC contributed to induced senescence and reduced adipogenesis (Spinelli et al. 2022).

### Cellular senescence in post-mitotic mature adipose cells

Even post-mitotic cells can assume a senescent phenotype, which has been shown in different cells, such as neurons, skeletal muscle fibers, cardiomyocytes and osteocytes, from aging mice (Tsiloulis and Watt 2015). In mature adipose cells from several mouse models, associated with age and obesity, a senescent phenotype has also been documented (Chen et al. 2015; Lee et al. 2022; Liu et al. 2018; Minamino et al. 2009; Vergoni et al. 2016), which recently has been confirmed in human mature adipose cells.

First and unexpectedly, Li et al. found that human subcutaneous mature adipose cells could activate a cell cycle program and that the cell cycle progression was associated with obesity and hyperinsulinemia (Li et al. 2021). Adipose cells from obese hyperinsulinemic individuals expressed G-specific cell cycle markers but not markers for the progression through mitosis, which is indicative of an endoreplicative or post-mitotic cell cycle (Li et al. 2021). This suggests that the cells get arrested in G_2_-phase there they exit the cell cycle and senesce. In a recent paper, we showed that human subcutaneous mature adipose cells assume a senescent phenotype in obese individuals, and this was even more pronounced in T2D individuals independent of BMI and age (Gustafson et al. 2022) (Fig. 2).γH2AX staining, as a measure of unresolved DNA damage response, the senescence marker *CDKN1A* as well as the expression of SASP factors (*IL6*, *CCL2*, *SERPINE1*, and *IL1B*) were increased in mature adipose cells from obese hyperinsulinemic individuals (Li et al. 2021). The senescence markers, *TP53, GLB1,* and *SERPINE1* were induced in mature adipose cells in obesity and even further increased in T2D and the level of senescence correlated to both degree of IR and adipose cell size. However, the insulin levels were not higher in T2D individuals compared to be equally obese non-diabetic individuals, which suggests that chronic hyperinsulinemia in not the only driver of CS in mature adipose cells. Furthermore, *ZMAT3* and *TNFA* were increased in T2D individuals compared to obese individuals of the same age (Gustafson et al. 2022). β-GAL, p16, plasminogen activator inhibitor 1 (PAI1, encoded by *SERPINE1*), p53 and the phosphorylation of mitogen-activated protein kinase 8 (also known as JNK1) were increased in mature adipose cells, which corresponded to decreased levels of adipogenic markers, PPARγ and GLUT4, and insulin sensitivity measured as serine 473 phosphorylation of AKT in T2D compared to lean individuals (Gustafson et al. 2022) (Fig. 2).

Moreover, high levels of cyclin D1 (encoded by *CCND1*), an important regulator of cell cycle progression, was detected in obese individuals with hyperinsulinemia (Li et al. 2021). This is in line with our findings of increased *CCND1* in mature adipose cells of obese individuals, which was further increased in T2D individuals (Gustafson et al. 2022). *CCND1* expression correlated also with cell size and the senescence markers *TP53*, *GLB1*, *SERPINE1* and *ZMAT3*. There was a gradual increase in cyclin D1, p16 and β-GAL in mature adipose cells comparing lean, obese and T2D individuals (Gustafson et al. 2022).

The chemotherapeutic agent Doxorubicin, like several other similar anti-cancer drugs, induces senescence in human APC as well as in differentiated cells with increased γH2AX, p21, ZMAT3 and cyclin D1 while the adipogenic markers PPARγ, adiponectin and fatty acid binding protein 4 (FABP4) are decreased as markers of de-differentiation of the cells (Gustafson et al. 2022).

### Cellular senescence in adipose tissue derived microvascular endothelial cells

Not only the storage of triglycerides in AT, which is a highly vascularized tissue, is crucial but also the transport of fatty acids through the microvascular endothelium is highly important for normal adipose tissue expansion. Endothelial cells (EC) can remain quiescent for years but, when needed, they become activated and initiate new vessel formation (angiogenesis), which is essential for the function of mature adipose cells as well as adipogenesis (Herold and Kalucka 2020). In obesity, AT is rapidly expanding and if the vascular network is not increased in parallel a dysfunctional AT will appear (Hammarstedt et al. 2018). Senescent EC have also been identified as important contributor to various metabolic and cardiovascular diseases (Bloom et al. 2023).

Interestingly, it was found that VAT-derived MVEC from obese individuals had a more pronounced senescent phenotype than SAT-derived MVEC together with a decreased expression of PPARγ (Villaret et al. 2010). It was also shown that PPARγ activity in human SAT-derived MVEC was regulated by senescence and that the senescent marker *CDKN2A* increased with aging. Furthermore, PPARγ turned out to be key regulator of fatty acid uptake in MVEC and *CDKN2A* showed a strong inverse correlation with the expression of the PPARγ target genes, *FABP4*, *CD36* and *solute carrier family 27 member 1* (*SLC27A1*) (Briot et al. 2018). Recently, we have shown that SAT-derived MVEC are highly specialized EC both regulating lipid transport and secreting lipids that activate PPARγ (Gogg et al. 2019). Most important is the crosstalk between MVEC and adipose cells in vivo to regulate the lipid transport and to activate PPARγ in APC to drive the adipogenic program. Senescence in MVEC seems not only to have impact on lipid handling and transport, but it also influences the ability of APC to differentiate with a dysfunctional AT and ectopic fat accumulation as consequences (Fig. 2).

## Conclusions and future possibilities

Senescent cells have left the cell cycle and become proinflammatory through secretion of SASP factors which include cytokines, chemokines, miRNA, DNA and other factors which can target ambient cells and induce senescence in these as well. Like many other cells, adipose tissue cells; both progenitor cells, mature adipose cells and microvascular endothelial cells, undergo senescence with aging but it is also increased with other common conditions like obesity and T2D. We only know some factors which can contribute to this including genetic factors and development of hyperinsulinemia. Adipose tissue cell senescence is associated with reduced adipogenesis, impaired adipose cell function, reduced PPARγ and reduced insulin sensitivity. Treating cell senescence in animal models improve many aging-associated conditions including insulin resistance, hyperglycemia and cardiovascular conditions. The current development of senomorphic agents may allow treatment of common aging-associated conditions in man as well but firm proof of this is currently not yet available.

## Abbreviations

AKT: Serine/threonine kinase 1
APC: Adipose tissue derived progenitor cells
AT: Adipose tissue
BCL: B-cell lymphoma
BMI: Body mass index
BMP4: Bone morphogenetic protein 4
*CCL2*: *C–C motif chemokine ligand 2*
CDK: Cyclin dependent kinase
CS: Cellular senescence
CVD: Cardiovascular disease
EC: Endothelial cells
FABP4: Fatty acid binding protein 4
FDR: First-degree relatives of type 2 diabetic
gH2AX: Phosphorylated H2A histone family X
*GLB1*: *Galactosidase beta 1*
IL: Interleukin
IR: Insulin resistance
MVEC: Microvascular EC
PPARγ: Peroxisome proliferator-activated receptor γ
RB: Retinoblastoma
SA-β-Gal: Senescence-associated β-galactosidase
SASP: Senescence-associated secretory phenotype
SAT: Subcutaneous adipose tissue
*SERPINE1*: *Serpin family E member 1*
T2D: Type 2 diabetes
TNFa: Tumor necrosis factor-α
*TP53*: *Tumor protein p53*
VAT: Visceral adipose tissue
ZNF423: Zinc finger protein 423
ZMAT3: Zinc finger matrin-type 3
